# Multimodal Retinal Vessel Analysis in CADASIL Patients

**DOI:** 10.1371/journal.pone.0112311

**Published:** 2014-11-05

**Authors:** Florian Alten, Jeremias Motte, Carina Ewering, Nani Osada, Christoph R. Clemens, Ella M. Kadas, Nicole Eter, Friedemann Paul, Martin Marziniak

**Affiliations:** 1 Department of Ophthalmology, University of Muenster Medical Center, Muenster, Germany; 2 Department of Neurology, University of Muenster Medical Center, Muenster, Germany; 3 Department of Medical Informatics and Biomathematics, University of Muenster, Muenster, Germany; 4 Department of Neurology, Charite University Medicine Berlin, Berlin, German; 5 NeuroCure Clinical Research Center, Berlin, Germany; Justus-Liebig-University Giessen, Germany

## Abstract

**Purpose:**

To further elucidate retinal findings and retinal vessel changes in Cerebral autosomal dominant arteriopathy with subcortical infarcts and leukoencephalopathy (CADASIL) patients by means of high resolution retinal imaging.

**Methods:**

28 eyes of fourteen CADASIL patients and an equal number of control subjects underwent confocal scanning laser ophthalmoscopy (cSLO), spectral-domain optical coherence tomography (SD-OCT), retinal nerve fibre layer (RNFL) measurements, fluorescein and indocyanine angiography. Three vessel measurement techniques were applied: RNFL thickness, a semiautomatic software tool based on cSLO images and manual vessel outlining based on SD-OCT.

**Results:**

Mean age of patients was 56.2±11.6 years. Arteriovenous nicking was present in 22 (78.6%) eyes and venous dilation in 24 (85.7%) eyes. Retinal volume and choroidal volume were 8.77±0.46 mm^3^ and 8.83±2.24 mm^3^. RNFL measurements showed a global increase of 105.2 µm (Control group: 98.4 µm; p = 0.015). Based on semi-automatic cSLO measurements, maximum diameters of arteries and veins were 102.5 µm (106.0 µm; p = 0.21) and 128.6 µm (124.4 µm; p = 0.27) respectively. Manual SD-OCT measurements revealed significantly increased mean arterial 138.7 µm (125.4 µm; p<0.001) and venous 160.0 µm (146.9; p = 0.003) outer diameters as well as mean arterial 27.4 µm (19.2 µm; p<0.001) and venous 18.3 µm (15.7 µm; p<0.001) wall thicknesses in CADASIL patients.

**Conclusions:**

The findings reflect current knowledge on pathophysiologic changes in vessel morphology in CADASIL patients. SD-OCT may serve as a complementary tool to diagnose and follow-up patients suffering from cerebral small-vessel diseases.

## Introduction

Cerebral autosomal dominant arteriopathy with subcortical infarcts and leukoencephalopathy (CADASIL) is a hereditary vascular small-vessel disease caused by Notch3 mutations [1]. It is distinguished from similar vascular disorders by the unique accumulation of granular osmiophilic material in systemic and particularly brain vasculature [2]. CADASIL represents a genetic model for small vessel diseases without the confounding factors of advanced age and other vascular diseases such as diabetes mellitus or arteriosclerosis. Besides migraine, transient ischemic attacks and strokes leading to severe disability and dementia in adult midlife in the absence of common vascular risk factors, CADASIL is clinically also characterized by various ophthalmologic findings [3]–[11]. Knowledge on the pathophysiology of CADASIL mainly derives from post mortem data due to the challenge of inspecting brain microvessels in vivo. Immunohistochemistry and electron microscopy have been proposed as useful diagnostic tools for CADASIL patients, yet, diagnostic sensitivity is still discussed controversially [12]–[14].

Architecture and properties of retinal blood vessels can provide essential clinical information not only in ocular disease but also in systemic disorders. Given the strong need for improved biomarkers in systemic vascular diseases, in-vivo imaging of retinal vessels appears to be a promising diagnostic approach. Recent technologic advances in imaging resolution and acquisition speed of commercial spectral-domain optical coherence tomography (SD-OCT) and confocal scanning laser ophthalmoscopy (cSLO) allow analysis of retinal morphology and retinal vessel morphology in greater detail and have contributed to our understanding of various retinal diseases. SD-OCT scans provide an ‘in-vivo histologic’ view and allow the differentiation of the various retinal layers as well as morphologic changes within these layers. Recently, high resolution SD-OCT proved to be capable of reliably measuring retinal vessel diameters and vessel wall thickness in vivo [15]–[17]. As CADASIL represents a model for small vessel diseases, this pathology is particularly suitable for research on vessel imaging. This study aims to evaluate three methods of retinal vessel analysis as well as to re-evaluate and to further elucidate retinal findings and retinal vessel changes in CADASIL patients using high resolution retinal imaging technology.

## Methods

### Demographics

Fourteen participants were recruited from the Department of Neurology at University of Muenster Medical Center. Diagnosis of CADASIL was confirmed in 12 patients by detection of Notch3 gene mutations and in two patients by vessel biopsy. Personal medical history was collected for each patient. Clinical investigations have been conducted according to the principles expressed in the Declaration of Helsinki. Informed consent, written and oral, have been obtained from the participants. The institutional review board of the ethics committee of the University of Berlin, Charité, approved the study.

All patients went through a test battery containing the Montreal Cognitive Assessment (MoCA), a questionnaire for migraine and vascular risk factors. The cut-off for a cognitive impairment was <26 points in the MoCA. The MoCA offers a high sensitivity for mild cognitive impairment (MCI) and therefore, it is well suited to detect early stages of dementia in CADASIL patients [18]. Common risk factors and co-morbidities for vascular diseases (smoking, hypertension, diabetes mellitus, overweight, coronary heart disease, arterial obstructive disease and stroke) were recorded. Furthermore, a detailed medical history for migraine was taken. A staging regarding the course of disease based on the classification by Verin et al. was performed [19]. Every patient was assigned to stage I through IV. Stage I includes common migraine episodes, abnormal MRI as well as a reliable diagnosis of CADASIL. Stage II includes psychiatric abnormality, transient ischemic attack while stage III requires history of stroke and dementia. Patients of stage IV show a progressed disease with severe dementia and frequent strokes.

All patients underwent a complete ophthalmic evaluation, including assessment of best-corrected visual acuity (BCVA), tonometry, slit-lamp biomicroscopy and fundus ophthalmoscopy. The visual field (VF) was defined by standard automated perimetry using a 30-2 central threshold test (Humphrey 740i, Zeiss Meditec, Germany). Heidelberg retina tomography (HRT, Heidelberg Engineering, Germany) was performed to rule out any glaucomatous damage. Age-matched healthy control subjects were attributed to CADASIL patients and underwent a complete ophthalmic evaluation as well.

### Imaging protocol

Color fundus photography (CFP) was performed according to standard 7-field-method (Visucam, Carl Zeiss Meditech, Germany). cSLO near infrared (IR) imaging (λ = 820 nm; Spectralis; Heidelberg Engineering, Germany) was performed with a minimum resolution of 768×768 pixels. The field of view was set at 30°×30° and centered on the macula as well as on the optic disc. Scans were saved for evaluation after 100 frames had been averaged using the automatic averaging and eye-tracking feature of the Spectralis device.

Imaging of the choroid was performed using enhanced depth imaging (EDI) OCT and indocyanine green angiography (ICG). EDI-OCT is a new approach to improve depth imaging and proved to be able to reliably image the full thickness of the choroid [20]. EDI-OCT volume scans were obtained consisting of 49 scans centered on the fovea. As previously described by Tanabe and colleagues, in each EDI-OCT scan, the cursor line marking the internal limiting membrane was moved manually to the outer border of the retinal pigment epithelium (RPE) [21]. Choroidal volume (CV) values in the posterior pole were obtained using the circular grid of the Early Treatment Diabetic Retinopathy Study (ETDRS) which is an integrated feature of the Heidelberg Eye Explorer software. This technique of manual CV measurements proved to be highly reproducible and repeatable and has a very small range of variability [22]. According to recorded EDI-OCT scans, additional SD-OCT volume scans were obtained to assess macular retinal volume (RV) within the ETDRS grid.

ICG allows for clearly displaying choroidal circulation and for identifying and delineating of choroidal watershed zones (CWZ) [23]. ICG was performed using 5 mg ICG dye (ICG-Pulsion, Pulsion Medical Systems, Germany) diluted in 5 ml aqueous solvent, injected into a peripheral vein in the arm. The field of view was set at 30° and centered on fovea. Recordings were performed according to the 7-field method. Excitation wavelength was at 787 nm and the range of transmitted light through the barrier filter was above 800 nm (Spectralis; Heidelberg Engineering, Germany). ICG of CADASIL patients were evaluated for the presence of CWZ as well as any other angiographic findings. Fluorescence angiography (FA) was performed in the same manner using an injection of 3 ml fluorescein (Fluorescein, Alcon, Germany). Excitation wavelength was at 488 nm and the range of transmitted light through the barrier filter was 500–700 nm (Spectralis; Heidelberg Engineering, Germany).

### Retinal nerve fiber layer measurements in SD-OCT

**Figure 1:**
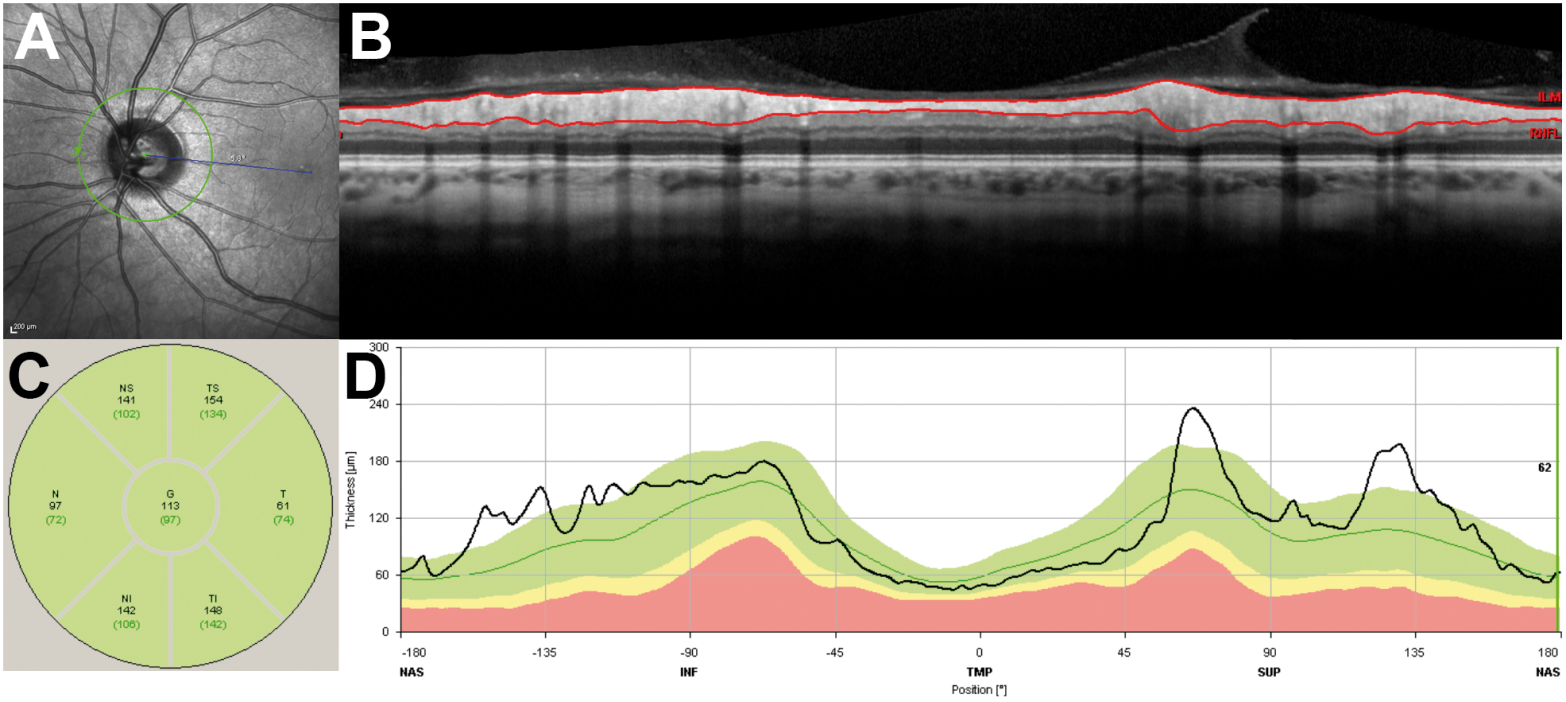
A–D Measurement of retinal nerve fiber layer (RNFL) thickness, using a circular B-scan placed around the optic disc. **A** Confocal scanning laser ophthalmoscopy infrared image shows optic disc with circular scan (diameter: 3.5 mm). **B** Corresponding spectral domain optical coherence tomography scan. Red marks delineate automatically borders of RNFL layer. **C** Presentation of RNFL thickness values for individual sectors and global measurement. **D** Individual measurement graphically compared to a healthy population.

Measurement of retinal nerve fiber layer (RNFL) thickness was performed using a circular B-scan placed around the optic disc (diameter: 3.5 mm) [24]. The exactly located placement around the center of the optic disc was carefully reviewed. Sectorial analysis of mean RNFL thickness is illustrated in Figure 1.

### Retinal vessel diameter measurements in cSLO

**Figure 2:**
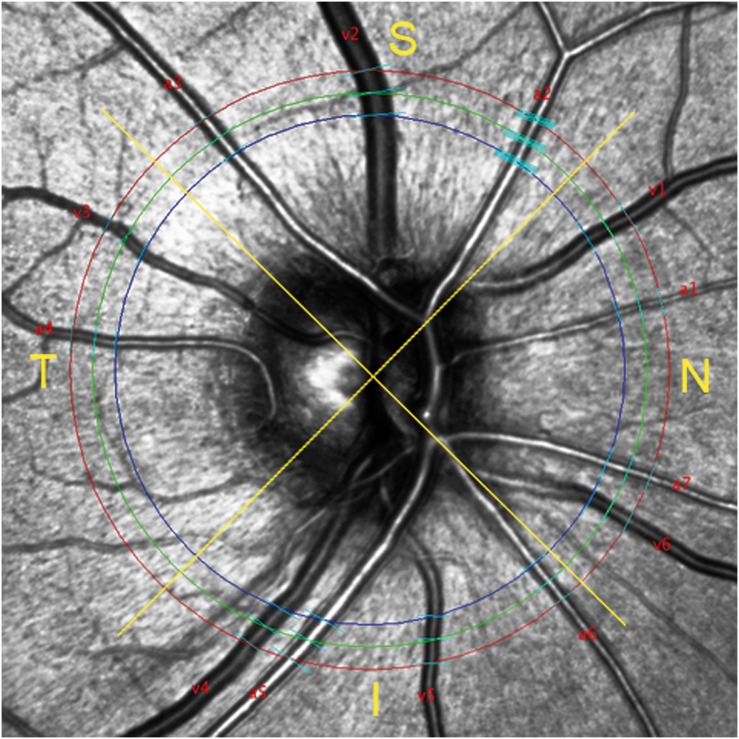
Confocal scanning laser (cSLO) infrared image illustrating semi-automatic measurement tool. Three concentrial circles (blue 3.2 mm, green 3.5 mm, red 3.8 mm) are placed around the optic disc. Vessel labelling marks arteries (a) and veins (v). Measurement lines (cyan) are defined by the software user. Additional measurement lines automatically produced by the software are shown exemplary in artery two (a2; set of five lines). Yellow lines separate superior (S), inferior (I), nasal (N) and temporal (T) quadrant.

Vessel identification in cSLO IR images was performed according to previously reported criteria [25]. Vessel measurements in native cSLO IR images were performed by a semi-automatic “Image-J”-Plugin. The Plugin creates three concentrial rings (diameter: small - 3.2 mm, medium - 3.5 mm, large - 3.8 mm) in the cSLO IR images (Figure 2). The medium ring lies exactly at the level of the circular SD-OCT scan used in RNFL measurement (diameter 3.5 mm). The other two rings of the semi-automated software lie slightly peripherally and centrally to the medium ring. The user labels each vessel as artery or vein and defines vessel measurement lines on all three rings. The software algorithm produces four additional measurements, two above and two below, based on the localization and orientation of each defined measurement point. In total, every vessel is measured at 15 different points. The rings are divided into four quadrants according to RNFL and SD-OCT measurements. A particular strength of the semi-automated technique based on cSLO is that vessel diameters are measured perpendicularly, while the applied SD-OCT scan crosses vessels tangentially according to the peripapillary circle.

cSLO IR images of retinal vessels show a central light reflex (CLR) and a dark vessel edge [25]. The software recognizes strong contrasts in reflectivity between outer vessel borders and surrounding retinal tissue, and between the CLR and inner vessel borders respectively (Figure 2). Changes in the vessel wall may alter the reflectivity in cSLO IR images. The reflex diameter may be interpreted as an indicator for the vessel architecture. Measurements were performed for the maximum measurable vessel diameter as well as for the reflex diameter. In both groups, artery-to-vein ratios (AVR) were calculated.

### Retinal vessel diameter and vessel wall thickness measurements in SD-OCT

**Figure 3:**
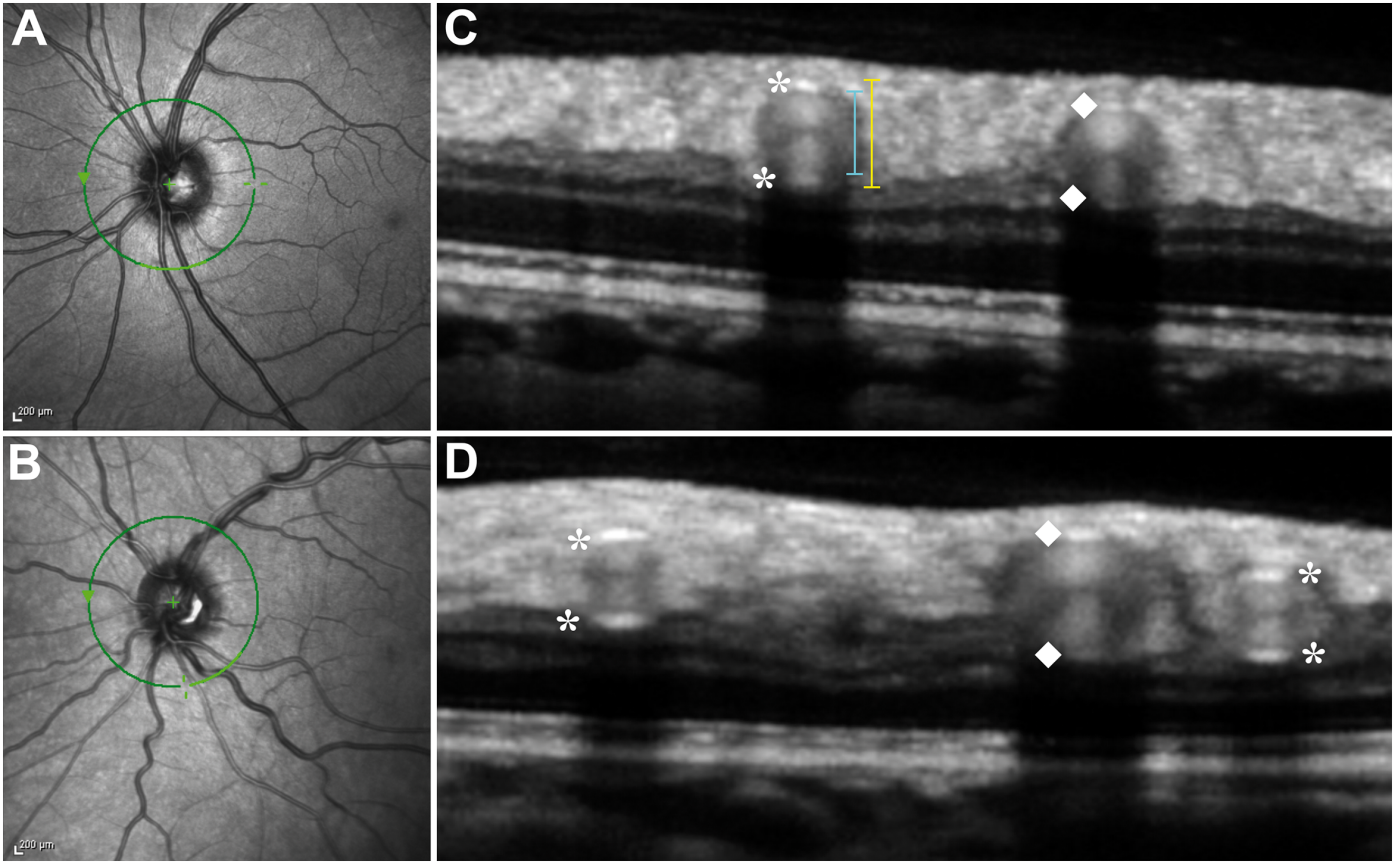
A–D Combined simultaneous confocal scanning laser ophthalmoscopy (cSLO) and spectral-domain optical coherence tomography (SD-OCT). **A–B** Infrared cSLO image centered on the optic disc of a healthy control subject (A) and a CADASIL patient (B). Green circle indicates the position of corresponding SD-OCT scan. Light green section inferiorly on the circle marks the localization of corresponding SD-OCT scan shown aside. **C–D** Magnified SD-OCT scans of healthy control subject (C) and CADASIL patient (D) show sections of major retinal vessels appearing as a group of heterogeneous reflectivities in a round-shaped configuration. Asterisks mark the inner and outer reflections of arterial vessel walls and diamonds indicate inner and outer reflections of venous vessel walls. Hyperreflectivities representing the vessel walls seem thicker and more accentuated in the CADASIL patient. Particularly in veins, demarcation of the inferior vessel wall (towards the retinal pigment epithelium) often remains challenging due to absorption effects also seen as acoustical shadow underneath the vessel (towards the retinal pigment epithelium). Note the typical hour-glass shaped configuration within the vessel lumen in both subjects. Lateral vessel walls cannot be visualized as OCT laser beam is not projected perpendicularly to them.

SD-OCT measurements of vessel diameter and vessel wall thickness around the optic disc proved to be highly reproducible and results correlate well with previous histologic studies [17], [18], [26]. Cross-sectional SD-OCT images reveal major retinal vessels as oval configurations with heterogeneous reflectivities, mainly in the RNFL and occasionally in the inner plexiform layer. Physiologic vessels show four distinctive hyperreflectivities. The top and bottom of the vessel walls correlate to the innermost and outermost hyperreflectivities. The arterial walls generally have higher reflectivity compared with the venous walls. Muraoka and colleagues previously reported that retinal vessels with physiologic blood flow show paired hyperreflectivities inside, which are frequently hourglass-shaped (Figure 3) [17]. According to this study, inner and outer vessel diameters of the four largest retinal arteries and veins were measured vertically by two blinded, independent readers (FA, JM) using the built-in manufacturer’s software (Heidelberg Eye Explorer) [17]. A mean value of the two readers as well as AVR values of outer and inner diameter were calculated in CADASIL patients and healthy controls.

Notably, the same circular SD-OCT scan centered on the optic disc that was used for RNFL analysis was used for manual SD-OCT vessel measurements. Moreover, this scan position is analogous to the medium size ring in cSLO IR vessel measurements. Thus, vessel diameter measurements in cSLO IR and SD-OCT as well as RNFL thickness measurements were performed exactly at the same anatomical localization warranting a thorough comparability of all three methods. As vessel diameters decrease as they run peripherally from the optic disk margin measurement localization is an important criterion [18].

### Statistical methods

HRT, ICG, FA and EDI-OCT volume scan was performed in the CADASIL group only while cSLO IR, SD-OCT volume scan and RNFL imaging was conducted in both groups. Both groups were homogeneous in age and gender. Statistical analysis was performed using IBM SPSS Statistics Ver. 22.0.0.0. Vessel diameter values were presented as mean ± standard deviation (SD). In cSLO IR images, all labelable and measureable vessels were used for analysis. Vessel diameters based on manual SD-OCT measurements were calculated using the four largest arteries and four largest veins. U-test and t-test were used to identify differences between control group and study group. A probability value of p<0.05 was considered indicative of statistical significance.

## Results

### Demographics

Mean age was 56.2±11.6 (control group: 54.5±10.1) years. Disease severity was as following: Stage I: 5 patients; St. II: 4 patients, St. III: 3 patients, St. IV: 2 patients. Two patients (14%) were clinically asymptomatic. Four (29%) had strokes in the medical history. The MoCA indicated dementia or MCI in 10 patients (71%). Four patients (29%) showed strong signs of dementia. A history of migraine was reported in 7 patients (50%), of whom one suffered from migraine with aura. Two (14%) of them reported a decrease of symptoms with increasing age.

BCVA was 0.75±0.25 given as decimal visual acuity values. Spherical equivalent was +0.27±1.39 diopters. One eye suffered from refractive amblyopia. Intraocular pressures were within normal limits. Slit lamp examination revealed no signs of iris atrophy as well as no signs of intraocular inflammation. One patient had a history of unilateral central retinal artery occlusion and showed panretinal laser coagulation spots with ghost vessels as well as an atrophy of the optic nerve head and was consequently excluded from RNFL and vessel analysis. One eye showed a drusen papilla that was excluded from RNFL evaluation. Two patients presented with macro papilla. No functional or morphological signs of glaucoma were present in any patient. Neuroretinal rim volume measured by HRT was 0.33±0.15 mm^3^. VF testing was performed in all patients except for one patient who was not capable of performing the examination due to his state of health. One patient showed scattered reduction of central and peripheral sensitivity secondary to his history of stroke. One examination was not evaluable due to fixation mistakes. The remaining VF tests were normal (–1.85±1.10 dB MD).

### Imaging

CFP and FA revealed neither cotton wool spots nor signs of ischemia in any eye. Sheathed arteries were detected in three eyes of two patients while no patient presented with tortuous arteries. No capillary leakage from blood vessels was seen during FA. Vessels with vascular sheathing also appeared normal during angiography. CFP further revealed arteriovenous nicking in 22 (78.6%) eyes and venous dilation in 24 (85.7%) eyes. RV in OCT volume measurements did not differ significantly between both groups (study group: 8.77±0.46 mm^3^; control group: 8.85±0.35 mm^3^; p = 0.341). CV in EDI-OCT volume scans was 8.83±2.24 mm^3^ in CADASIL patients. ICG revealed a CWZ in 6 (42.9%) CADASIL patients. Otherwise, there was no evidence for choroidal vascular occlusion or hypoperfusion.

### Retinal nerve fiber layer measurements in SD-OCT

Mean number of averaging frames was 91.2±17.4. Both groups showed a regular retinal layer configuration. Global RNFL measurements revealed significant differences between both groups (Study group: 105.2 µm; control group: 98.4 µm; p = 0.015). Consequently, a clear significance towards a thicker RNFL in CADASIL patients is evident. (Table 1).

**Table 1:** Retinal nerve fiber layer thickness (RNFL) measured by spectral-domain optical coherence tomography: healthy controls compared to CADASIL patients.

Peripapillary sector	Mean RNFL thickness [µm ± SD]				
	healthy controls		CADASIL		p-value
	n = 28		n = 23		
**nasal sup.**	115.3	±26.4	121.7	±25.2	0.368
**nasal**	75.9	±13.1	85.3	±13.8	0.027
**nasal inf.**	105.1	±28.3	116.2	±24.1	0.173
**temporal inf.**	138.1	±19.9	143.2	±18.3	0.289
**temporal**	69.9	±9.4	73.0	±8.2	0.254
**temporal sup.**	136.5	±20.8	143.3	±21.6	0.244
**superior** *	125.9	±18.5	132.5	±19.6	0.349
**inferior** *	121.6	±20.4	129.7	±16.4	0.08
***global***	*98.4*	*±10.5*	*105.2*	*±9.5*	*0.015*

(n) number of eyes.

*superior and inferior measurements were calculated based on data from nasal superior and temporal superior quadrants and from nasal inferior and temporal inferior quadrants respectively.

### Retinal vessel diameter measurements in cSLO

In the study group, maximum diameters of arteries and veins were 102.5 µm (Control group: 106.0 µm; p = 0.21) and 128.6 µm (124.4 µm; p = 0.27) respectively. Inner reflex diameters of arteries and veins were 32.2 µm (Control group: 30.7 µm; p = 0.13) and 37.2 µm (35.7 µm; p = 0.23) (Table 2). AVR of all vessels based on the maximum diameter was 0.80 for CADASIL patients and 0.85 for healthy controls.

**Table 2:** Semi-automated vessel measurements based on confocal scanning laser ophthalmoscopy (cSLO): healthy controls compared to CADASIL patients.

Vessel	Peripapillarysector				Mean [µm ± SD]			
		healthy controls			CADASIL			p-value
		n = 28		v	n = 25		v	
**Arteries**								
Max diameter	nasal	94.16	±20.33	53	88.95	±20.96	51	0.201
	superior	109.98	±29.32	66	110.43	±24.94	63	0.926
	temporal	89.03	±21.39	11	85.03	±21.56	16	0.639
	inferior	112.55	±27.99	60	109.48	±27.39	60	0.545
	*global*	*105.99*	*±27.92*	190	102.49	*±26.74*	190	0.213
Inner reflexdiameter	nasal	25.68	±10.54	40	27.93	±8.23	43	0.280
	superior	32.10	±8.00	64	34.47	±8.26	61	0.106
	temporal	26.25	±10.03	11	27.01	±8.15	14	0.836
	inferior	33.22	±8.44	55	34.53	±9.25	54	0.441
	*global*	*30.68*	*±9.42*	170	32.19	*±9.17*	172	0.134
**Veins**								
Max diameter	nasal	111.33	±19.07	30	103.93	±20.32	35	0.137
	superior	123.02	±27.26	66	133.61	±36.28	58	0.067
	temporal	81.34	±4.96	1	54.42	±1.93	2	n/a
	inferior	132.62	±31.02	51	139.98	±36.44	50	0.277
	*global*	*124.38*	*±28.74*	148	128.61	*±36.60*	145	0.272
Inner reflexdiameter	nasal	32.66	±7.07	21	31.26	±8.80	26	0.558
	superior	34.80	±8.29	63	37.88	±10.47	51	0.082
	temporal	25.21	±2.83	1	2.83	±5.11	1	n/a
	inferior	38.45	±7.77	47	40.80	±11.64	48	0.251
	*global*	*35.74*	*±8.22*	132	37.22	*±11.53*	126	0.235

(n) number of total eyes; (v) number of total vessels; (n/a) too few vessels in the respective sector to calculate p-value.

### Retinal vessel diameter and vessel wall thickness measurements in SD-OCT

High quality SD-OCT images are crucial to perform accurate vessel diameter and vessel wall thickness measurements. A clear demarcation was required between inner vessel wall and vessel lumen as well as outer vessel wall and retinal tissue. Due to questionable border discrimination of vessel walls, two arteries and three veins were excluded in the healthy control group as well as three veins in the study group. At vessel crossing points and in case of close vicinity of vessels, SD-OCT offers better vessel border discrimination compared to cSLO. Modification of contrast in SD-OCT scans slightly improved border discrimination particularly in arteries.

Mean arterial and venous outer diameters were 138.7 µm (Control group: 125.38 µm; p<0.001) and 160.0 µm (146.9 µm; p = 0.003). Mean inner arterial and venous diameters were 84.0 µm (87.0 µm; p = 0.238) and 123.4 µm (115.5 µm; p = 0.05). Vessel wall thickness was calculated as difference between outer and inner vessel diameter measurements. Mean wall thickness was 27.4 µm (19.2 µm; p<0.001) in arteries and 18.3 µm (15.7 µm; p<0.001) in veins (Table 3). AVR values of inner and outer diameters of CADASIL patients were AVR_out_ 0.87, AVR_in_ 0.68 and for healthy controls AVR_out_ 0.85, AVR_in_ 0.75.

**Table 3:** Manual vessel measurements using spectral-domain optical coherence tomography (SD-OCT): healthy controls compared to CADASIL patients.

Vessel	Mean [µm±SD]					
		healthy controls		CADASIL		p-value
		n = 28		n = 25		
**Arteries**	Outer diameter	125.38	±19.42	138.71	±20.25	<0.001
	Inner diameter	87.02	±17.06	83.99	±18.76	0.238
	Vessel wall	19.18	±3.03	27.36	±4.47	<0.001
**Veins**	Outer diameter	146.92	±25.72	159.95	±32.97	0.003
	Inner diameter	115.47	±24.38	123.35	±30.63	0.05
	Vessel wall	15.73	±2.99	18.30	±4.91	<0.001

(n) number of eyes.

## Discussion

CADASIL has gained increasing interest as a model for the more common forms of ischemic cerebral small-artery diseases and subcortical ischemic vascular dementia [27]. CADASIL is characterized by a thickening of the arterial wall leading to lumen stenosis, the presence of a non-amyloid granular osmiophilic material within the media extending into the adventitia, as well as morphological alterations of smooth-muscle cells [28], [29]. Cerebral and retinal arterioles share a similar anatomy, physiology, and embryology and there is evidence for an association between retinal vessel changes and cerebral small vessel disease [30], [31]. The aim of our study was to benefit from recent advances in in-vivo retinal imaging and to analyze and re-evaluate previously reported retinal findings and retinal vessel changes in CADASIL patients. To our knowledge, this is the first study to apply these refined in-vivo imaging tools to CADASIL patients and to report detected changes in vessel architecture due to this pathology.

Functional data as well as funduscopic findings like vascular sheathing, arteriovenous nicking and venous dilation are in line with previous reports [3]–[12]. The prevalence of single findings may vary as different study groups are certainly heterogeneous regarding the clinical stage of included patients and usually contain only a limited number of patients.

Previous histologic data revealed that ocular vessel pathologies in CADASIL patients are limited to retinal vessels only, while choroidal vessels are unaffected [5]. For the first time, we report in-vivo imaging data on the choroid of CADASIL patients that clinically confirm former histologic findings. Neither ICG nor EDI-OCT imaging revealed pathologic findings in the study group. The CV as well as the number of CWZ are in line with previously reported data of healthy probands [23], [32], [33].

RNFL measurements in CADASIL patients based on previous generation time-domain OCT instruments consistently showed a significant reduction in peripapillary RNFL thickness [6]–[8]. SD-OCT allows a refined RNFL analysis around the optic disc with a differentiated eight sector analysis grid. Interestingly, our measurements revealed an increased global RNFL thickness in CADASIL patients. As retinal vessels run within the RNFL for the most part, this finding suggests that a pathologic thickening of peripapillary retinal vessels in CADASIL patients, as seen histopathologically, may result in an increase of global RNFL thickness [2]. Considering the anatomic distribution of peripapillary vessel trunks one may consequently hypothesize that this finding must be particularly found in the vessel rich superior and inferior sectors potentially serving as an additional marker of vessel alteration in CADASIL patients. Yet, a significant increase in those vessel rich sectors was not found for supporting this thesis. An increase in RNFL thickness in CADASIL patients certainly is an interesting finding, and it might be interpreted as a result of vessel thickening. However, previous studies on RNFL measurements in CADASIL patients report a decrease in RNFL thickness [6]–[8]. These contradictory results suggest that RNFL measurements currently do not appear suitable as screening or follow-up tool in this patient group and require further research.

Fischer et al. previously described the challenge of visualizing and measuring outer vessel diameters in native cSLO IR images as borders between retinal vessels and surrounding tissue often become indistinct. Similarly to funduscopy, the authors postulate that native cSLO IR only captures inner vessel diameter, while vessel outer diameters as well as vessel walls remain undetectable [34].

We found no significant changes in the arterial and venous maximum diameter in CADASIL patients using native cSLO IR imaging. Based on the hypothesis by Fischer and colleagues, these measurements can be interpreted as inner vessel diameter. Previous studies controversially discussed the value of retinal vessel characteristics such as AVR representing factors for assessing vascular status or even risk assessment. Ikram and co-workers reported that increased retinal venous calibers are associated with stroke and progression of cerebral small vessel disease [35], [36].

As CADASIL patients represent a high risk group for stroke, a venous dilatation in retinal vessels could be expected in these patients. Contrary, Chui et al. recently postulated that vessel walls are detectable using adaptive optics cSLO [37]. Additionally, our data shows AVR values higher than 0.8 in cSLO IR imaging in both groups, which gives rise to doubts whether cSLO IR in fact measures inner vessel diameters considering that AVR values of about 2/3 for the inner vessel diameter were reported previously [34], [38]. We use the term ‘maximum vessel diameter’ for the diameter measured in cSLO IR images. Regardless of the question whether cSLO records the inner or outer diameter, we did not find significant changes in the maximum diameters between healthy controls and CADASIL patients suggesting that pathologic structural vessel changes in CADASIL are not accessible to native cSLO IR imaging.

Goldenberg et al recently proposed a non-invasive, in-vivo method for measuring retinal vessel caliber based on SD-OCT [18]. Furthermore, Muraoka and colleagues proved that measuring retinal vessel walls in healthy subjects and in patients is reliable using a manual measurement tool in SD-OCT [16], [17]. A paired, frequently hourglass-shaped hyperreflectivity inside the vessels was observed in healthy subjects and interpreted as result of physiologic blood flow. As previously described, this pattern is substantially altered in patients suffering from retinal vein occlusion [16]. In our study group as well as in our control group, the hourglass-shaped hyperreflectivity was consistently seen suggesting that blood flow is not severely disturbed in major retinal vessels of CADASIL patients (Figure 3).

Manual vessel measurements using SD-OCT revealed that the outer diameter in arteries and veins in CADASIL patients was highly significantly thicker than in healthy subjects. Moreover, the vessel wall was highly significantly thicker in both venous and arterial vessels. An important difference between arteries and veins is the inner diameter. In veins, the inner diameter i.e. the lumen showed significantly higher values in CADASIL subjects. The inner diameter of arteries in CADASIL patients did not reveal a significant difference, yet, the arterial lumina tended to narrow. If thickening of arteries in CADASIL patients affects lumen diameters is still subject of debate. Dong et al recently confirmed a substantial thickening of leptomeningeal arteries of CADASIL patients, which is primarily a result of distinct intimal hyperplasia that does not affect lumen diameter [39]. AVR values of healthy controls revealed a considerable difference between outer and inner diameters suggesting that unlike cSLO IR, SD-OCT allows for differentiating between outer and inner diameters. In 2006, Roine et al reported significantly lower AVR values (0.53) in 33 CADASIL patients compared to healthy controls (0.61) based on fundus photography, which approximately corresponds to our inner diameter AVR values based on manual SD-OCT [10]. This additionally supports the notion that fundus photography shows inner diameters, while cSLO IR does not.

Measurements of our healthy control group are in accordance with recently reported data [16], [17]. In summary, using manual SD-OCT measurements CADASIL patients revealed a distinct difference in inner and outer diameter as well as in vessel wall thickness in both arteries and veins not only compared to our control group but also to measurements of larger healthy populations from the literature (Table 4).

**Table 4:** Data overview regarding manual retinal vessel measurements based on spectral-domain optical coherence tomography.

Vessel	Mean [µm ± SD]								
		Goldberg et al*		Muraoka et al#		healthy controls#		CADASIL#	
		n = 29		n = 238		n = 14		n = 14	
**Arteries**	Outer diameter	127.8	±13.4	122.5	±13.1	125.38	±19.42	138.71	±20.25
	Inner diameter			87.8	±9.4	87.02	±17.06	83.99	±18.76
	Vessel wall			17.4	±2.4	19.18	±3.03	27.36	±4.47
**Veins**	Outer diameter	145.3	±15	141	± 13.1	146.92	± 25.72	159.95	±32.97
	Inner diameter			113.7	±12.5	115.47	± 24.38	123.35	±30.63
	Vessel wall			13.7	±2.1	15.73	± 2.99	18.30	±4.91

(n) number of patients.

*measurements performed at 960 µm from the optic disc edge.

# circular SD-OCT scan 3.5 mm in diameter.

Manual vessel measurements in peripapillary SD-OCT scans allow for in-vivo identification of vessel walls, outer and inner diameter of retinal arteries and veins in CADASIL patients. This technique visualizes morphologic changes in vessel architecture reflecting histologic and pathophysiologic knowledge on this disease.

Obviously, the small number of subjects included in the study precludes any definitive interpretation. Yet, CADASIL is a rare disease and in those patients included diagnosis was confirmed by genetic testing and vessel biopsy. Furthermore, the clinical stage of the disease was heterogeneous within the study group. As automated software is not commercially available, retinal vessel diameters had to be measured manually on SD-OCT sections. Study results must be interpreted cautiously bearing in mind that SD-OCT does allow for highlighting differences of reflectivity within the human retina in-vivo, however, no strict correlations with histology have been demonstrated yet. So far it remains unclear, for instance, which vessel wall layer or which property of the vessel wall results in hyper- or hyporeflectivities seen in SD-OCT. Therefore, one cannot assume for sure that the identified and measured reflectivities in this study in fact represent the entire vessel wall. Furthermore, all depicted vessels within an SD-OCT scan are captured by the laser beam in various angles, which may lead to altered reflection properties of each individual vessel wall. Finally, absorption effects of the inner vessel tissue (towards the vitreous) may cause a challenging demarcation of outer vessel walls (towards the RPE) particularly in venous vessels since veins exhibit a weaker reflectivity signal compared to arteries due to their different wall architecture. These facts certainly limit the validity of morphologic changes in vessel wall thickness observed in SD-OCT.

Regarding continuous improvements in retinal imaging, retinal vessel analysis may become more relevant not only in ophthalmologic but also in systemic and neurologic diseases [40]. In the near future, adaptive optics SLO appears to be the next step in in-vivo retinal imaging as it is increasingly capable of non-invasively detecting and monitoring morphological changes within retinal vascular wall morphology [37]. Image acquisition using adaptive optics systems and the subsequent image processing is extremely time consuming, which currently limits widespread clinical application.

This is the first study to report retinal findings and retinal vessel measurements in CADASIL patients based on high resolution imaging. CADASIL patients revealed a thicker RNFL caused by enlarged vessel diameters. Increased retinal venous lumina, a known risk factor for stroke, were found in manual SD-OCT measurements. Thickened vessel walls as found in manual SD-OCT measurements correspond to previous histologic reports. Finally, reduced arterial vessel lumina as shown in SD-OCT represent the ischemic component of this disease.

In the future, retinal imaging will certainly not replace MRI in CADASIL patients as it is indispensable for detection of cerebral damage as well as for differential diagnosis. Nevertheless, besides MRI, genetic diagnostic and immunohistology, high resolution retinal vessel imaging may be accounted as a complementary tool to diagnose and follow-up CADASIL patients and other cerebral small-vessel diseases in the future.
